# Early Identification of Cognitive Impairment in Community Environments Through Modeling Subtle Inconsistencies in Questionnaire Responses: Machine Learning Model Development and Validation

**DOI:** 10.2196/54335

**Published:** 2024-11-13

**Authors:** Hongxin Gao, Stefan Schneider, Raymond Hernandez, Jenny Harris, Danny Maupin, Doerte U Junghaenel, Arie Kapteyn, Arthur Stone, Elizabeth Zelinski, Erik Meijer, Pey-Jiuan Lee, Bart Orriens, Haomiao Jin

**Affiliations:** 1 School of Health Sciences University of Surrey Guildford United Kingdom; 2 Center for Self-Report Science University of Southern California Los Angeles, CA United States; 3 Department of Psychology University of Southern California Los Angeles, CA United States; 4 Leonard Davis School of Gerontology University of Southern California Los Angeles, CA United States; 5 Center for Economic and Social Research University of Southern California Los Angeles, CA United States

**Keywords:** machine learning, artificial intelligence, cognitive impairments, surveys and questionnaires, community health services, public health, early identification, elder care, dementia

## Abstract

**Background:**

The underdiagnosis of cognitive impairment hinders timely intervention of dementia. Health professionals working in the community play a critical role in the early detection of cognitive impairment, yet still face several challenges such as a lack of suitable tools, necessary training, and potential stigmatization.

**Objective:**

This study explored a novel application integrating psychometric methods with data science techniques to model subtle inconsistencies in questionnaire response data for early identification of cognitive impairment in community environments.

**Methods:**

This study analyzed questionnaire response data from participants aged 50 years and older in the Health and Retirement Study (waves 8-9, n=12,942). Predictors included low-quality response indices generated using the graded response model from four brief questionnaires (optimism, hopelessness, purpose in life, and life satisfaction) assessing aspects of overall well-being, a focus of health professionals in communities. The primary and supplemental predicted outcomes were current cognitive impairment derived from a validated criterion and dementia or mortality in the next ten years. Seven predictive models were trained, and the performance of these models was evaluated and compared.

**Results:**

The multilayer perceptron exhibited the best performance in predicting current cognitive impairment. In the selected four questionnaires, the area under curve values for identifying current cognitive impairment ranged from 0.63 to 0.66 and was improved to 0.71 to 0.74 when combining the low-quality response indices with age and gender for prediction. We set the threshold for assessing cognitive impairment risk in the tool based on the ratio of underdiagnosis costs to overdiagnosis costs, and a ratio of 4 was used as the default choice. Furthermore, the tool outperformed the efficiency of age or health-based screening strategies for identifying individuals at high risk for cognitive impairment, particularly in the 50- to 59-year and 60- to 69-year age groups. The tool is available on a portal website for the public to access freely.

**Conclusions:**

We developed a novel prediction tool that integrates psychometric methods with data science to facilitate “passive or backend” cognitive impairment assessments in community settings, aiming to promote early cognitive impairment detection. This tool simplifies the cognitive impairment assessment process, making it more adaptable and reducing burdens. Our approach also presents a new perspective for using questionnaire data: leveraging, rather than dismissing, low-quality data.

## Introduction

### Background

Cognitive impairment encompasses both mild cognitive impairment (MCI) and dementia. MCI is considered to be an intermediate state between normal aging and dementia [1] and may initially not interfere with daily life but potentially progress to dementia and cause significant functional impairment [2]. Recent biomedical research continues to yield novel treatments, such as lecanemab (Eisai and Biogen) and Donanemab (Eli Lilly and Company) [3,4], which can effectively slow cognitive decline if applied at its early stages (MCI or mild dementia). This underscores the importance of detecting cognitive impairment early for the cognitive health of older adults.

Nevertheless, cognitive impairment underdiagnosis remains a concerning issue around the world. In the United States, cognitive impairment assessment is included in Medicare’s Annual Wellness Visit [5], but less than 20% of older adults use this preventive service [6,7]. In countries such as the United Kingdom, routine cognitive impairment assessment is not recommended for all older adults [8]. This underuse or lack of provision of preventive cognitive impairment assessment, combined with challenges faced by primary care providers in identifying early cognitive impairment on top of managing other health issues, often leads to cognitive impairment underdiagnosis [9,10].

Considering these challenges, health professionals working in community environments such as community health and social workers (CHSWs) have become increasingly important in cognitive health management for older adults. CHSWs primarily focus on promoting overall well-being within community settings, such as nonprofit organizations, government agencies, and community centers, and often work with specific populations such as older adults. The World Health Organization’s Global Action Plan has made dementia a priority for public health action, accelerating a paradigm shift in the prevention and care of cognitive impairment from clinical systems toward family and community-based services [9]. This shift emphasizes the essential roles of CHSWs in identifying and referring older adults with early cognitive impairment symptoms to clinical care providers for further assessment and treatment.

Although CHSWs have a growing role in cognitive health management, several challenges may hinder the implementation of timely cognitive impairment assessment in community environments. Administering a cognitive impairment assessment, even with a so-called “brief” tool such as the Telephone Interview for Cognitive Status (which consists of 11 items and takes about 10 minutes with a trained administrator), is easier said than done [11]. Given the broad range of responsibilities of CHSWs, integrating cognitive impairment assessments into their existing tasks could stretch their capacity and limit the time available for other essential services. Besides, CHSWs may encounter challenges related to a lack of specific training and expertise in cognitive health assessment, because their educational background often focuses on social work and public health. This knowledge gap could decrease their ability to accurately identify symptoms of cognitive impairment and confidently refer individuals for further clinical evaluation. Finally, stigma and cultural barriers surrounding cognitive impairment may also present challenges for CHSWs [12]. Older adults or their families might be hesitant to disclose cognitive impairment symptoms within community settings, leading to inaccurate results of cognitive impairment assessment [12].

### Low-Quality Response in Questionnaires

In conducting surveys or questionnaires, researchers usually expect respondents to answer questions honestly and accurately. However, some respondents might lack the cognitive capability to think carefully and answer each question genuinely, leading to a lower quality of response [13-16]. The definition of low-quality response (LQR) in this paper is relatively neutral and is not motivated by intentional deception, but rather by invalid, unreliable, or erroneous responses [17,18]. Manifestations of the LQR are diverse [19-21], including but not limited to (1) skipping questions—respondents may selectively answer and bypass certain queries; (2) contradictory answers—for similar questions, respondents might provide conflicting responses; (3) oversimplified responses—for instance, always choosing “agree” or “do not know” for all questions; and (4) inaccurate or unreliable answers. This could be due to the respondents not fully understanding the question or lacking the time or motivation to ponder over it.

Completing a questionnaire is a task that requires multiple cognitive functions working in synergy [22], such as attention, working memory, and executive functions. Respondents with subtle cognitive deficits might resort to suboptimal answering strategies to cope with the psychological demands of responding to questions [17,23]. Some studies have found that older adults with cognitive deficits may exhibit stronger signals of LQR during questionnaires [19-21,24]. For instance, they might more frequently skip questions or opt for the “do not know” answer. Early cognitive decline might first manifest in complex tasks in the daily life of people, among which, completing a questionnaire is one.

### Gaps in Research

Recent research on early identification of cognitive impairment has been using advanced computational statistics techniques such as machine learning to develop predictive models of cognitive impairment [25-28]. Nevertheless, challenges persist in effectively implementing such cognitive impairment predictive models within community environments. Existing studies on machine learning and cognitive impairment classification mostly used clinical biomarkers such as magnetic resonance imaging and cerebrospinal fluid analysis as predictors [29-31]. These approaches are not feasible in community settings due to their reliance on high-cost or invasive methods for obtaining predictor information. Furthermore, most current studies on machine learning and cognitive impairment classification have been based on small clinical samples (sample size typically ranged from 140-550), where resampling or case-controlling techniques were widely used to derive analytic samples with balanced cognitive impairment and noncognitive impairment proportions [32-34]. The proportion of cognitive impairment cases in such datasets can be significantly higher than the estimated prevalence (about 15%-20%) observed among older adults in communities [35]. While these resampling and case-controlling techniques may enhance the model’s sensitivity to the minority class (ie, the cognitive impairment) [36,37], existing research has shown that reliance on these techniques can lead to discrepancies in the distribution of data and introduce model bias [38,39].

It is worth noting that recent studies have started to explore the use of large community-based samples with questionnaire data to develop machine learning models for cognitive impairment classification [40-43]. However, there remain several important gaps in this genre of research. First, issues related to LQRs, such as nonresponse, extreme answers, and acquiescence, are ubiquitous in the questionnaire data of older adults [19,44-46]. Direct use of questionnaire response data without proper handling of the potential LQR issue may reduce the validity of the prediction models. Second, the number of predictors used in these studies was generally substantial (approximately 22-44), which can make rapid data collection and cognitive impairment assessment challenging. Lastly, widely used machine learning techniques such as regularization are not designed to produce unbiased predictions, because bias-variance trade-off is an essential component of these techniques [47]. Consequently, the predicted probability from these models does not necessarily reflect the true likelihood of cognitive impairment. This poses a challenge for end users, such as CHSWs, in selecting an appropriate threshold. Existing studies often fall short in providing guidance on determining this threshold.

### This Study

This study aimed to develop a novel machine learning tool for the early identification of cognitive impairment in community settings using psychometric indices generated from subtle inconsistencies in questionnaire responses. A distinctive feature of this study is that rather than filtering out the original questionnaire responses due to potential LQR issues (eg, eliminating low-quality answers), we relied on LQR indices, derived from psychometric methods, as predictors in our machine learning models to classify cognitive impairment. In community environments, CHSWs routinely administer brief questionnaires to identify well-being issues [48], generating a rich amount of questionnaire response data despite that the contents of these questionnaires are usually not directly related to assessing cognitive impairment. This study will investigate whether the relationships between LQR and cognitive impairment could be exploited to develop a machine learning tool for early cognitive impairment identification.

## Methods

### Study Design

**Figure 1:**
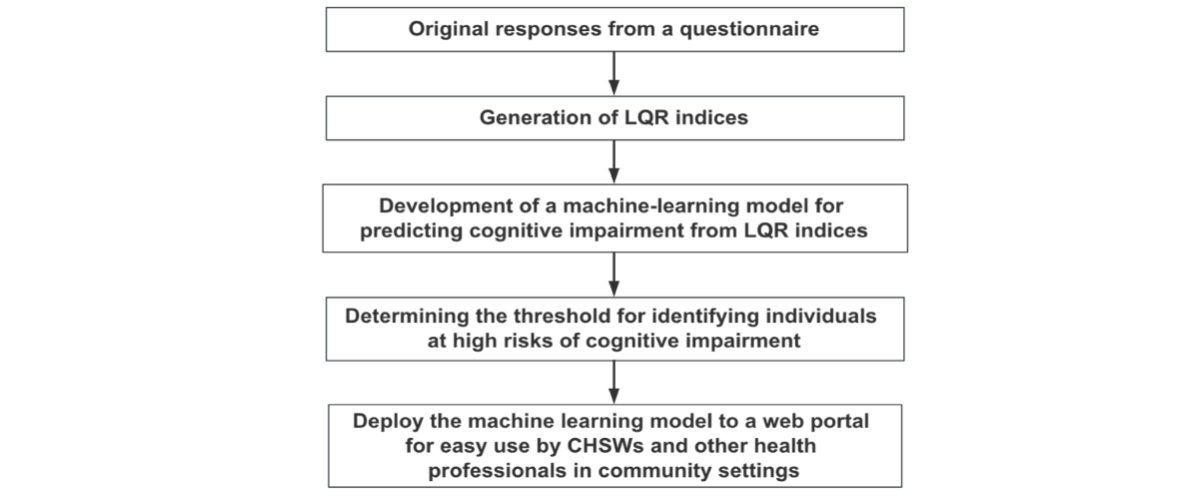
Overview of the workflow and methods for the proposed tool development. CHSW: community health and social worker; LQR: low-quality response.

An overview of the methodology adopted in this study is provided in Figure 1. We followed the guidelines outlined in the TRIPOD (Transparent Reporting of a Multivariable Prediction Model for Individual Prognosis or Diagnosis) statement for reporting the development and validation of the multivariate predictive models (Multimedia Appendix 1) [49].

### Study Sample

Development of the machine learning tool was based on individuals aged 50 years and older from the Health and Retirement Study (HRS), a community-based longitudinal aging study involving a representative sample of older adults in the United States [50]. As a part of this study, demographics including race and ethnicity are collected using surveys. In 2006 (wave 8) and 2008 (wave 9), the HRS administered the leave-behind survey, which explored various aspects of participants’ psychological well-being and life experiences [51,52]. A random half (n=8681, 51.13%) of participants completed the survey in 2006, and the remaining participants (n=8296, 48.87%) completed it in 2008. Our analysis included participants from the leave-behind survey, excluding those whose questionnaires were completed by proxy, those with missing responses to all scale items, or those lacking key follow-up records (such as survival status). This resulted in a total of 12,942 individuals included in our analysis. While our analysis examined all 21 available questionnaires in the HRS leave-behind survey (Multimedia Appendix 2) [51], 4 questionnaires, that is, the optimism, hopelessness, life purpose, and life satisfaction scales, were used as the primary data source to develop the machine learning tool. These questionnaires were brief and assessed aspects that are closely related to the common focus of CHSWs on overall well-being as a positive psychological attitude, satisfaction with life, clear life goals, and optimism significantly contribute to enhancing the quality of life, extending the anticipated lifespan, and reducing mortality risk in those aged 50 years or older [53-56].

### LQR Indices

To model subtle inconsistencies in questionnaire responses as predictors in our machine learning model, we created two types of LQR indices using psychometric methods. For each questionnaire, we first fitted a graded response model—a common psychometric model based on the item response theory—to the data to obtain an estimate of the respondents’ “latent trait” score [57]. We did this to remove information related to content (eg, people’s optimism levels) and to isolate statistically “misfitting” response patterns indicative of LQR regardless of question content. The first index, the squared residuals, was then derived by calculating the squared differences between the observed questionnaire item responses and the statistically expected responses given a respondent’s latent trait [58]. Larger differences between observed and expected responses indicate severe problems with LQR. The second index was defined as the probability of a respondent choosing the observed response given their latent trait [59]. Selecting statistically fewer probable responses can indicate problems with LQR. The LQR indices were generated using R (version 4.2.2; R Foundation), mirt (version 1.38.1; York University), and tidyverse (version 2.0.0; RStudio) [59,60].

### Predicted Outcomes

Cognitive impairment status was ascertained using the validated criteria developed by Langa et al [61] for the HRS. In brief, a cognitive status score was calculated from cognitive tests of immediate and delayed word recall, an attention and working memory task (serial sevens), and counting backward from 20. The total score has a range from 0 to 27 with higher scores indicating better cognitive performance. Thresholds of dementia (0-6 points), cognitively impaired with no dementia (7-11 points), and cognitively normal (12-27 points) were applied [61]. The cognitively impaired with no dementia and dementia categories were then combined into a single cognitive impairment category to indicate whether the individual currently has cognitive impairment. To investigate the longitudinal prognostic utility of the LQR indices, we also created a secondary predicted outcome in the supplemental analysis, that is, dementia or mortality in the next 10 years, which was derived from the HRS follow-up records.

### Model Development and Training

Using the LQR indices derived from each questionnaire, we trained and compared seven machine learning models for the binary classification task of forecasting cognitive impairment. Specifically, we used the multilayer perceptron (MLP) technique. MLP is a feed-forward neural network that is structured with input, hidden, and output layers [62]. Compared to conventional machine learning, it possesses robust adaptive learning capabilities, enabling the automatic extraction of abstract information from raw predictor variables without the need for labor-intensive manual transformations [62]. Moreover, the MLP is particularly adept at discerning nonlinear relationships between predictors and outcomes [63], facilitating the modeling of potential complex associations between the LQR indices and cognitive impairment. In contrast to certain advanced hybrid deep learning models, the MLP boasts a lighter architecture and parameter setup, which reduces computational burdens [64]. In addition to training the models using only LQR indices, another version of the models was developed using the LQR indices in combination with two easily obtained demographic variables, that is, age and gender, as predictors.

The dataset had 1.25% missing values (3898 of 310,608 data points), with 1499 of 12,942 (11.58%) participants having at least one missing item. These missing values were imputed using a regression-based iterative imputation method (details in Multimedia Appendix 3) [65]. The imputed data was then split into training (n=9059, 70%) and testing sets (n=3883, 30%) using stratified random sampling to ensure a consistent cognitive impairment prevalence (n=1641, 18.11% for the training set and n=704, 18.1% for the testing set) across both sets. We used batch normalization and class weighting strategies during the training process [66,67]. This allowed our model to support raw data input without scaling and resampling steps.

The MLP model comprised four hidden layers with a rectified linear unit activation function [68]. To reduce overfitting, we added dropout layers after each hidden layer [69]. The output layer used a sigmoid activation function with an initialization strategy to accelerate model convergence [68,70]. During the training phase, we used 10-fold cross-validation on the training sets [71]. Model weights and biases were adjusted based on the binary cross-entropy loss. The data were further split into 80% (n=7247) training subsets and 20% (n=1812) validation subsets in each fold. The area under the curve (AUC) was monitored on the validation subset to guide mechanisms such as early stopping [72]. We also used the Adam optimizer with a dynamic learning rate to optimize the training process, ensuring that only the best-performing model was retained for the final evaluation of the test dataset [66,73]. Hyperparameter selection was initially guided by KerasTuner (Google) [74]. Based on its recommended values, extensive iterative experiments were conducted to ascertain the selected configurations, as listed in Multimedia Appendix 4. The model training was carried out using Python (version 3.9.16; Python Software Foundation), TensorFlow (version 2.12.0; Google, Google Brain), and scikit-learn (version 1.2.1; French Institute for Research in Computer Science and Automation) [66,75,76]

We compared the performance of the MLP to 6 different predictive models. These included 2 classical machine learning models (logistic regression and decision trees) [77,78], 2 ensemble learning models (XGBoost and LightGBM) [79,80], and 2 hybrid deep learning models bidirectional-gated recurrent unit and convolutional neural network-long short-term memory [81-83]. We compared bidirectional-gated recurrent unit and convolutional neural network-long short-term memory models due to the potential dependencies among LQR indices. The LQR indicators are derived from multi-item questionnaires, where multiple items are designed to load onto a single latent factor. Consequently, there may be correlations between LQR indices within items from the same questionnaires. For the classical machine learning and ensemble learning models, we used grid search to determine the optimal hyperparameters. In the case of the hybrid deep learning models, we adopt the same training approach as the MLP and a similar architecture for the fully connected layers. All models were trained by 10-fold cross-validation and evaluated on the same unseen test dataset.

### Threshold Determination

We determined the threshold for cognitive impairment risk scores predicted by the model based on the ratio of underdiagnosis cost to overdiagnosis cost, aiming to minimize the total cost of underdiagnosis and overdiagnosis [84]. Underdiagnosis refers to when the assessment tool identifies an individual with cognitive impairment as cognitively normal, while overdiagnosis refers to when the tool identifies a cognitively normal individual as having cognitive impairment. When applying the cognitive impairment assessment tool in community settings, individuals whose predicted risk scores are higher than the threshold would be referred to follow-up clinical cognitive impairment assessment. Thus, the cost of overdiagnosis would be equal to the cost of conducting one follow-up clinical cognitive impairment assessment. The cost of underdiagnosis would be more profound, including the cost of not receiving timely clinical assessment and treatment of cognitive impairment. Users of the cognitive impairment assessment tool may choose different cost ratios tailored to the needs of their practices. A larger cost ratio would be appropriate when the follow-up clinical assessment and treatment of cognitive impairment is readily available. With the emergence of effective medications such as lecanemab and donanemab [3,4], this cost ratio would more likely to become larger in the future.

### Evaluation of Model Performance and Efficiency

Predictive accuracy of the assessment tool was evaluated using the AUC value. AUC values range from 0 to 1, with a higher score indicating better predictive performance [72]. Moreover, the efficiency of using the tool in identifying individuals at high risk of cognitive impairment was evaluated using an efficiency curve approach. The first step was to calculate the proportion of individuals referred to further clinical assessment for cognitive impairment based on predicted results from the tool, which served as a proxy measure of the resources required to conduct follow-up clinical assessment and treatment. Then, the proportion of individuals with cognitive impairment identified by the follow-up clinical assessments was calculated, which acted as a proxy measure for the desired outcome of the tool’s implementation and subsequent cognitive impairment assessment. These proportions were computed at different predicted risk score thresholds from the tool, and the paired proportion values were plotted on a curve. The tool’s efficiency was then compared with plausible rule-based screening strategies, which included conducting cognitive impairment assessments for all individuals aged 65 years and older, or those with cardiovascular diseases such as high blood pressure, heart diseases, possible stroke or transient ischemic attack, or diabetes that is one of major risk factors for cardiovascular disease. This approach of comparing screening efficiency based on proxy measures of needed resources and desired outcomes has been previously implemented by research to compare machine learning–based assessment against rule-based screening strategies [85].

### Ethical Considerations

This study is approved by the University of Southern California institutional review board (UP-22-00147) and the University of Surrey Research Integrity and Governance Office (FHMS 21-22 216 EGA).

## Results

Sample characteristics are described in Table 1. In addition, Multimedia Appendix 5 presents sample characterization of the training and testing sets. There were 9059 individuals in the training dataset with an average age of 68.9 (range 50-104.6, SD 9.85) years, of which 18.11% (n=1641) had cognitive impairment according to the Langa-Weir criteria. The testing set included 3883 individuals. The average age of the testing sample was 68.8 (range 50-100.7, SD 9.72) years and 18.1% (n=704) in the testing set had current cognitive impairment. The comparison between training and testing sets showed no significant differences.

**Table 1:** Sample characteristics (N=12,942).

Variables			Participants, n (%)
**Age (years)**			
	50-59	2613 (20.19)	
	60-69	4113 (31.87)	
	70-79	3935 (30.4)	
	80 and older	1938 (14.97)	
**Gender**			
	Female	7357 (56.85)	
	Male	5242 (40.5)	
**Marital status**			
	Married	8112 (62.68)	
	Not married	4486 (34.66)	
**Race**			
	African American	1511 (11.68)	
	White	10,588 (81.81)	
	Other	500 (3.86)	
**Ethnicity**			
	Hispanic	903 (6.98)	
	Not Hispanic	11,696 (90.37)	
**Education level**			
	High school and below	6833 (52.8)	
	Some college	2918 (22.55)	
	College graduate and above	2847 (22)	
**Self-reported diseases**			
	High blood pressure	7029 (54.31)	
	Diabetes	2402 (18.56)	
	Heart disease	2963 (22.89)	
	Possible stroke or transient ischemic attack	947 (7.32)	
	With 2 or more of the above diseases	3728 (28.81)	

The MLP has the highest predictive performance on the testing dataset compared to other predictive methods. As shown in Multimedia Appendix 6, when using the LQR indices as predictors, the MLP ranked first or tied for first in AUC values for 16 of the 21 questionnaires. After including age and gender as additional predictors, the MLP ranked first or tied for first in AUC for 18 of the 21 questionnaires. As a result, we chose the MLP as our predictive model. In addition, we evaluated the performance of using raw questionnaire responses as predictors in logistic regression models as baseline performance. Among the 21 questionnaires, the baseline model’s AUC ranged from 0.47 to 0.64, with a mean of 0.56 (SD 0.04) and a median of 0.55 (IQR 0.04). The performance of using raw questionnaire responses for cognitive impairment prediction slightly outperformed random guessing (AUC=0.5), but was inferior to using LQR indices for prediction in our machine learning models.

**Figure 2:**
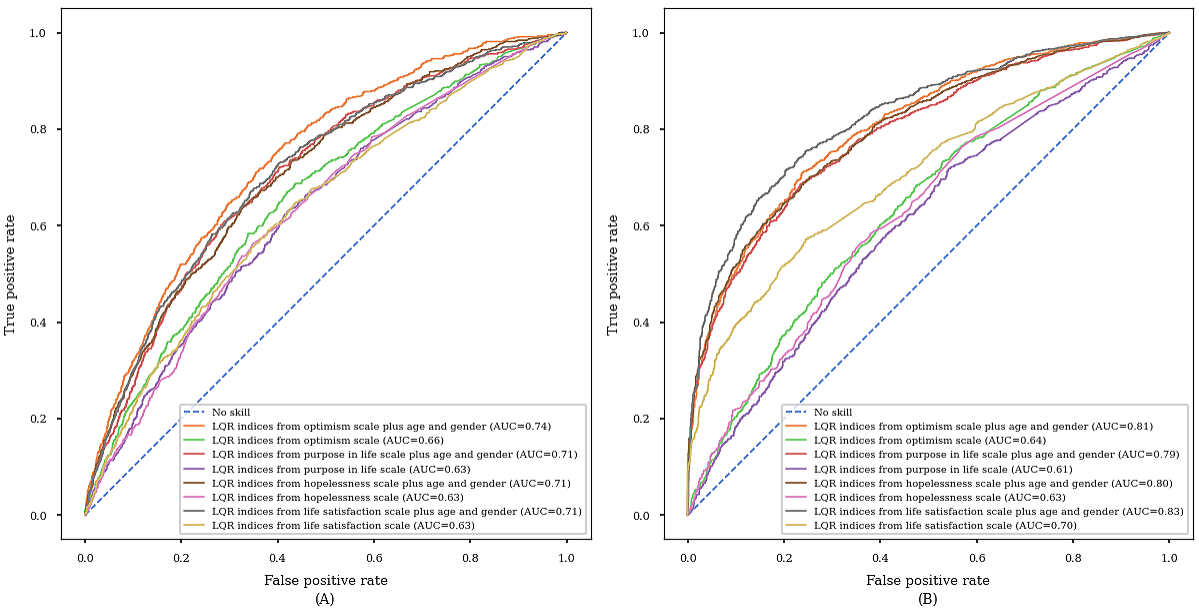
(A) AUC curves for predicting current cognitive impairment and (B) AUC curves for predicting dementia or mortality in the next 10 years. AUC: area under the curve.

Predictive accuracy of the MLP models is summarized in Multimedia Appendix 7 and AUC curves are shown in Figure 2. The AUC values ranged from 0.63 to 0.66 across the 4 questionnaires when using LQR indices as predictors. The AUC values were improved to the range of 0.71 to 0.74 when age and gender were included as additional predictors. The AUCs were similar across the 4 questionnaires, and no evident signs of overfitting were observed during the training and validation phases (Multimedia Appendix 8). The LQR indices derived from the optimism scale performed the best among all 21 available questionnaires (Multimedia Appendix 6). The AUC for predicting dementia or mortality in the next ten years ranged from 0.61 to 0.70 and improved to 0.80 to 0.83 when age and gender were included as additional predictors.

Additionally, the evaluation of different thresholds revealed that as the cost ratio of underdiagnosis to overdiagnosis of cognitive impairment increased, the proportions of individuals referred for further assessment and the identification of cognitive impairment cases also increased. The results demonstrated that the performance differences between models with and without age and gender as additional predictors became smaller as the cost ratio increased. For instance, with a cost ratio of underdiagnosis to overdiagnosis of two, the model using LQR indices from the optimism scale resulted in 3% (n=116) of all individuals being referred for further clinical cognitive impairment assessment and 7% (n=49) of all cognitive impairment cases being identified. On the other hand, the model using LQR indices from the same scale plus age and gender as predictors led to 10% (n=388) of individuals referred for further assessment and 24% (n=169) of cognitive impairment cases identified. When the cost ratio increased to four, the model using LQR indices alone resulted in 39.99% (n=1533) of individuals being referred and 59.9% (n=422) of cognitive impairment cases being identified, and the model using LQR indices plus age and gender led to 35% (n=1359) of individuals being referred and 63.1% (n=444) of cognitive impairment identified. As mentioned above, the advancements in dementia treatment imply increased cost ratios of underdiagnosis to overdiagnosis, suggesting that the thresholds corresponding to larger cost ratios are more relevant for future practices. In light of this, a cost ratio of 4 was used as the default choice for the tool, because it provides a reasonable trade-off between underdiagnosis and overdiagnosis.

**Figure 3:**
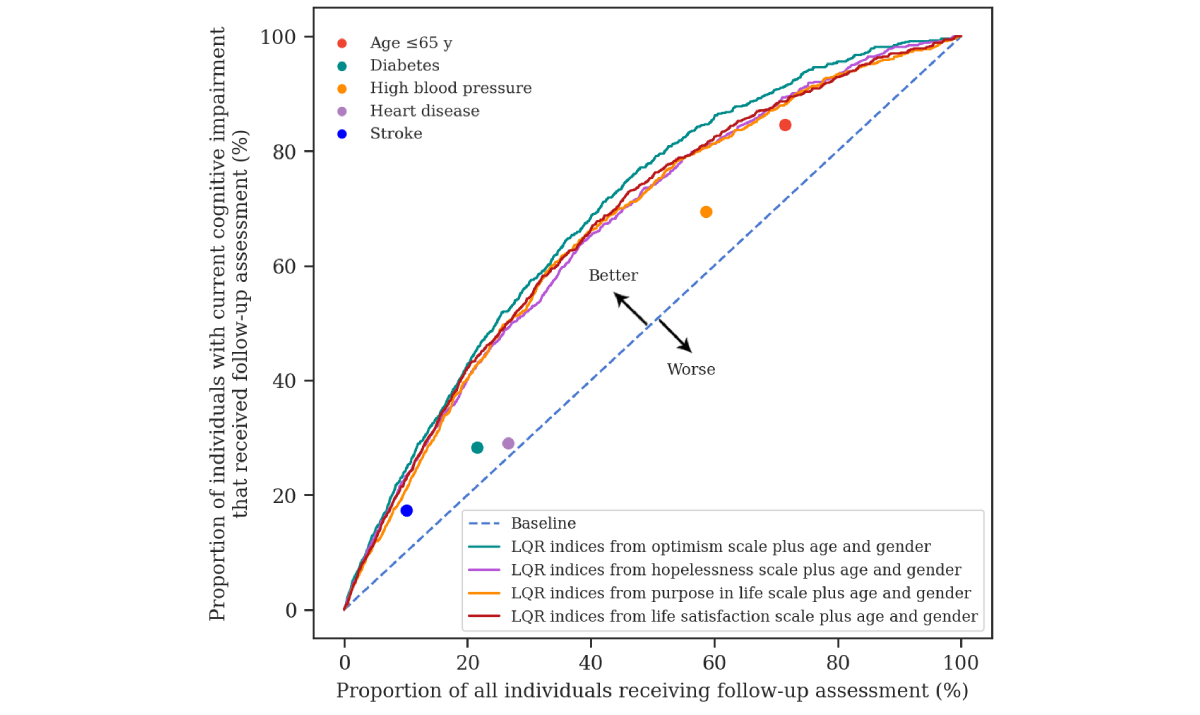
Comparison of machine-learning-based and rule-based identification efficiency for high-risk individuals with cognitive impairment.

Compared to rule-based screening strategies, the cognitive impairment assessment tool showed greater efficiency in identifying individuals at high risk of cognitive impairment. As shown in Figure 3, the efficiency curves corresponding to the four questionnaires were all located to the left of rule-based strategies, indicating higher efficiency as fewer resources were required to achieve the same desired outcome. Moreover, the tool offered greater flexibility as users could choose thresholds depending on the needs of their practices. We selected model thresholds at a default cost ratio of 4 for comparison between the two strategies, as shown in Table 2. Except for the rule “high blood pressure,” where the screening efficiency is relatively balanced, the screening efficiency under the other rules is generally polarized, making it difficult to reach a reasonable trade-off. For instance, although the rule “age ≥65” years leads to 84.9% (n=598) of this study’s sample with cognitive impairment being identified, it also consumes the most resources (n=2757, 71% of the sample will need to receive follow-up assessments), almost twice as much as the machine learning-based screening strategies. The machine learning methods are more efficient in identifying individuals with cognitive impairment. For instance, in the model based on the optimism scale, 35% (n=1359) of the sample needs to receive follow-up assessments to ensure that 63.1% (n=444) of individuals with cognitive impairment are identified. The proportion receiving follow-up assessments is approximately 51% and 41% less compared to the “age ≥65” years and “high blood pressure” rules, respectively, yet it achieves an output (ie, the proportion of individuals with cognitive impairment identified) that is approximately 75% and 91% of theirs. This indicates that machine learning approaches are more efficient per resource usage and capable of achieving a higher identification ratio with less resource input.

Moreover, we explored the performance of LQR indices as predictors across different age groups, and the results are presented in Table 3. Overall, the cognitive impairment assessment tool demonstrated greater predictive accuracy and efficiency in the 50- to 59-year and 60- to 69-year age groups. Notably, in the optimism scale, the LQR indices consistently exhibited the best predictive performance (AUC=0.72) in the 50- to 59-year age group, regardless of whether age and gender were included as predictors. The tool performed better in predicting cognitive impairment among relatively younger older adults, suggesting that LQR indices may be more sensitive to early cognitive deficits.

**Figure 4:**
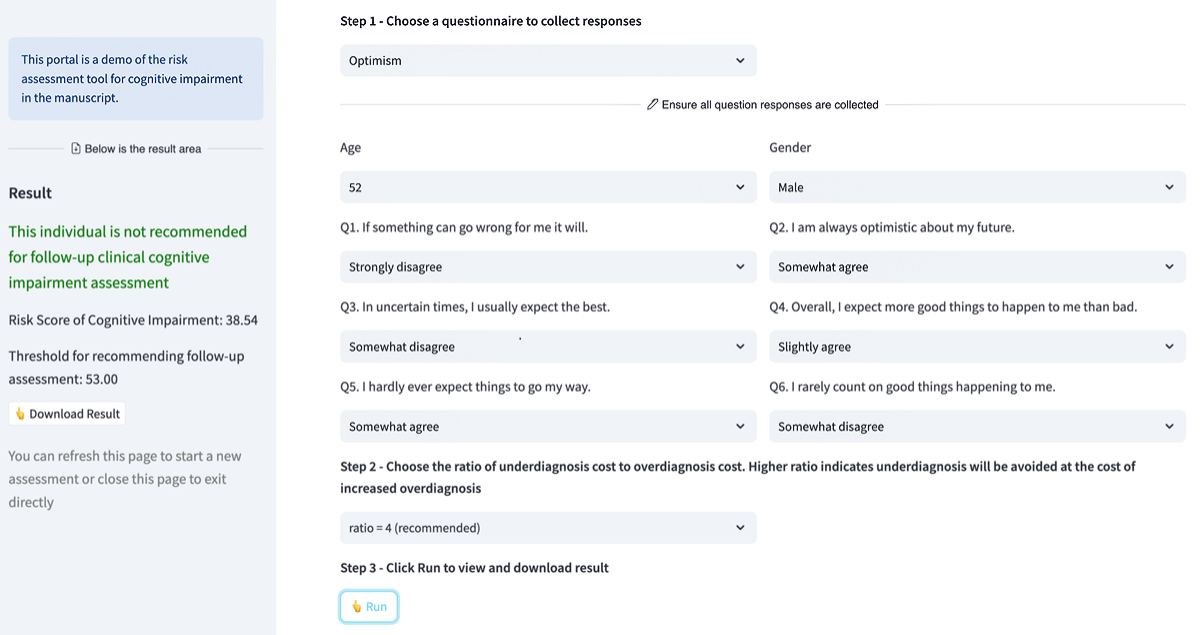
A web portal allowing free public use of the machine-learning tool for cognitive impairment assessment based on subtle inconsistencies in questionnaire responses.

Finally, the cognitive impairment assessment tool was deployed to a web portal for free public access (URL available in the reference list [86]). As shown in Figure 4, the tool allows users to enter responses from one of the four questionnaires examined in this study, set up thresholds by choosing a cost ratio (default is 4), predict risk scores of cognitive impairment, and generate recommendations for whether follow-up cognitive health assessment would be suggested. Users can choose a different cost ratio based on their circumstances. The tool can also be expanded using the same modelling approach described in this paper to incorporate additional questionnaires in the future.

**Table 2:** Comparison of identification efficiency for high-risk individuals with cognitive impairment between machine learning–based and rule-based approaches under a cost ratio of 4.

Rule or model name	Proportion of all individuals receiving follow-up assessment, n（%）	Proportion of individuals with current cognitive impairment that received follow-up assessment, n (%)
Age ≥65 years	2757 (71)	598 (84.9)
Diabetes	854 (22)	197 (28)
High blood pressure	2291 (59)	486 (69)
Heart disease	1048 (26.99)	204 (29)
Stroke	388 (10)	120 (17)
LQR^a^ indices from optimism scale plus age and gender	1359 (35)	444 (63.1)
LQR indices from purpose in life scale plus age and gender	1281 (32.99)	422 (59.9)
LQR indices from hopelessness scale plus age and gender	1553 (39.99)	458 (65.1)
LQR indices from life satisfaction scale plus age and gender	1243 (32.01)	408 (58)

^a^LQR: low-quality response.

**Table 3:** Comparison of performance and efficiency of the LQR^a^ indices as predictors for predicting current cognitive impairment across scales and age groups under a cost ratio of 4.

Models and age groups (years)			AUC^b^		Sample, n (%)		Sample with cognitive impairment, n (%)		Individuals receiving follow-up assessment, n（%）		Individuals with current cognitive impairment that received follow-up assessment, n (%)
**LQR indices from optimism scale plus age and gender**											
	50-59	0.72		790 (20.4)		73 (9)		65 (8)		22 (30)	
	60-69	0.7		1268 (32.66)		154 (12.2)		214 (16.9)		51 (33)	
	70-79	0.66		1256 (32.35)		257 (20.5)		588 (46.8)		170 (66.2)	
	80 and above	0.62		569 (14.7)		220 (38.7)		529 (93)		210 (95.5)	
**LQR indices from purpose in life scale plus age and gender**											
	50-59	0.64		790 (20.4)		73 (9)		41 (5)		9 (12)	
	60-69	0.66		1268 (32.66)		154 (12.2)		167 (13.2)		48 (31)	
	70-79	0.61		1256 (32.35)		257 (20.5)		562 (44.8)		153 (59.5)	
	80 and above	0.56		569 (14.7)		220 (38.7)		533 (93.7)		212 (96.4)	
**LQR indices from hopelessness scale plus age and gender**											
	50-59	0.62		790 (20.4)		73 (9)		87 (11)		13 (18)	
	60-69	0.65		1268 (32.66)		154 (12.2)		193 (15.2)		42 (27)	
	70-79	0.62		1256 (32.35)		257 (20.5)		693 (55.2)		184 (71.6)	
	80 and above	0.61		569 (14.7)		220 (38.7)		566 (99.5)		218 (99.1)	
**LQR indices from life satisfaction scale plus age and gender**											
	50-59	0.63		790 (20.4)		73 (9)		37 (5)		9 (12)	
	60-69	0.64		1268 (32.66)		154 (12.2)		151 (11.9)		33 (21)	
	70-79	0.66		1256 (32.35)		257 (20.5)		516 (41.1)		156 (60.7)	
	80 and above	0.57		569 (14.7)		220 (38.7)		549 (96.4)		212 (96.4)	
**LQR indices from optimism scale**											
	50-59	0.72		790 (20.4)		73 (9)		309 (39.1)		53 (73)	
	60-69	0.71		1268 (32.66)		154 (12.2)		489 (38.6)		104 (67.5)	
	70-79	0.62		1256 (32.35)		257 (20.5)		527 (42.0)		139 (54.1)	
	80 and above	0.59		569 (14.7)		220 (38.7)		277 (48.7)		128 (58.2)	
**LQR indices from purpose in life scale**											
	50-59	0.67		790 (20.4)		73 (9)		321 (40.6)		47 (64)	
	60-69	0.65		1268 (32.66)		154 (12.2)		508 (40.1)		91 (59)	
	70-79	0.6		1256 (32.35)		257 (20.5)		568 (45.2)		151 (58.8)	
	80 and above	0.54		569 (14.7)		220 (38.7)		346 (60.8)		145 (65.9)	
**LQR indices from hopelessness scale**											
	50-59	0.65		790 (20.4)		73 (9)		261 (33)		38 (52)	
	60-69	0.67		1268 (32.66)		154 (12.2)		404 (31.9)		83 (54)	
	70-79	0.6		1256 (32.35)		257 (20.5)		433 (34.5)		120 (46.7)	
	80 and above	0.6		569 (14.7)		220 (38.7)		245 (43.1)		116 (52.7)	
**LQR indices from life satisfaction scale**											
	50-59	0.66		790 (20.4)		73 (9)		291 (36.8)		42 (58)	
	60-69	0.64		1268 (32.66)		154 (12.2)		507 (40)		89 (58)	
	70-79	0.65		1256 (32.35)		257 (20.5)		541 (43.1)		159 (61.9)	
	80 and above	0.58		569 (14.7)		220 (38.7)		276 (48.5)		121 (55)	

^a^LQR: low-quality response.

^b^AUC: area under the curve.

## Discussion

### Principal Findings

In this study, we developed a novel machine learning tool for predicting cognitive impairment based on LQR indices derived from subtle inconsistencies in questionnaire responses. One unique advantage of the tool is its reliance solely on the brief questionnaire that does not directly assess cognitive impairment. Of all the questionnaires evaluated, the best performer was the optimism scale, which comprises just six questions. Crucially, the content of these questionnaires does not need to be directly related to cognitive impairment. This flexibility allows health professionals, such as CHSWs, to select questionnaires that resonate more with their practical emphasis, such as focusing on overall well-being aspects such as optimism, hopelessness, life purpose, and life satisfaction [53-56]. Powered by our machine learning models, these questionnaires now enable a “passive or backend” cognitive impairment assessment with acceptable accuracy (AUC around 0.7) for prescreening and screening purposes. This approach alleviates the need for health professionals such as CHSWs to undergo specialized cognitive impairment assessment training and addresses potential stigmatization concerns. Residents can engage without fearing labels such as “potentially cognitively impaired” or being seen as “likely to develop dementia” from receiving cognitive impairment evaluations [12]. In essence, our tool streamlines the cognitive impairment assessment process, making it more adaptable and less burdensome for both professionals and the community.

Compared with existing machine learning models for cognitive impairment prediction, our machine learning model is developed from a large epidemiological sample without using class-balancing techniques. This ensures that the proportion of cognitive impairment in both training and testing sets mirrors the actual prevalence of cognitive impairment in communities. As mentioned above, our model demands fewer efforts in gathering data on predictors compared to its counterparts. The tool also allows for flexible threshold selection based on the ratio of costs related to underdiagnosis to the costs related to overdiagnosis. Such flexibility facilitates end users to navigate the trade-off between underdiagnosis and overdiagnosis when using a risk assessment tool.

Compared to clinical studies using clinical biomarkers as predictors for dementia [26,29], our tool still has a gap in predictive performance. However, our goal does not intend to replace existing clinical cognitive impairment assessment tests or tools. Instead, we hope that the tool we have developed will lower the threshold for the usage of cognitive impairment risk assessment, thereby adapting it to the needs of community settings. The tool may establish an effective bridge between the community end and the clinical end, allowing the clinical end to align resources more productively, resulting in fewer unnecessary inputs and greater efficiency in the health care system. In addition, by comparing to recent studies that use similar HRS data to predict cognitive impairment [43,87,88], our tool demonstrated predictive performance close to those studies, even though other studies used clinical or cognitive impairment-related risk factors as additional predictors. For instance, a study achieved an AUC of 0.78 but used 13 additional cognitive impairment risk factors (eg, race, stroke history, and glycated hemoglobin) alongside questionnaire data [87].

Finally, our approach expands the boundary of current research by innovatively integrating knowledge and techniques from data sciences and psychometrics to enhance the cognitive health of older adults. Central to our methodology is the machine learning tool rooted in LQR indices that are designed to detect latent cognitive deficits through modelling subtle inconsistencies in questionnaire responses. Unlike clinical markers of cognitive impairment [29-31], these psychometric indices are not only cheaper but also simpler to obtain, making them ideal for community settings. More importantly, the psychometric method allows for the evaluation of cognitive impairment independently of the questionnaire’s content. Further, while conventional research methodologies typically handle low-quality data by either eliminating or statistically adjusting them by methods such as weighting [89-92], our approach presents a new perspective: leveraging, rather than dismissing, low-quality data. This strategy underscores the potential of our methodology to enhance data utility for future research.

In addition to the development and validation of this tool, we further explored the possibility of improving predictive performance to provide insights for future research. We experimented with two approaches: one was to incorporate education level as an additional predictor, and the other was to combine four selected scales. The results showed that both methods could enhance the prediction performance of current cognitive impairment using LQR as predictors (Multimedia Appendix 9). It is worth pointing out that the number of items in each questionnaire had no direct impact on the predictive effect (Multimedia Appendix 6), but combining different questionnaires did improve the discriminative ability of the model. The application of these two approaches may increase the burden of assessment as more predictors are added to the model. Furthermore, incorporating other indicators of LQR, such as prolonged response time, may increase the predictive accuracy [93].

This study is constrained by the questionnaires available in the HRS, potentially limiting its generalizability to other questionnaire response data. However, an ongoing comprehensive meta-analysis of longitudinal aging surveys from ten countries indicates that the relationships between LQR indices and cognitive functioning remain consistent across various questionnaires and nations [94]. Additionally, ascertainment of cognitive impairment in this study is based on empirically established thresholds rather than detailed clinical evaluations. Clinical diagnosis of cognitive impairment is intricate, often requiring a mix of cognitive assessments, health assessments, laboratory tests, and brain imaging. Given this complexity, large aging survey studies such as the HRS find it impractical to clinically evaluate every participant. Consequently, these studies typically perform clinical evaluations for a select group and then use empirically derived standards, such as the Langa et al [61], to determine cognitive impairment in more extensive samples. Lastly, this study is limited by its nature as a secondary data analysis. Future research should implement the tool in real-life settings and conduct external validation and impact assessment to ensure its effectiveness.

### Conclusions

The machine learning tool developed in this study provides a novel yet practical solution for tackling the challenges of early identifying cognitive impairment in community environments. The approach adopted in this study innovatively integrates psychometric methods with data science techniques and large questionnaire response data, resulting in a risk assessment tool that can facilitate health professionals working in community environments to conduct “passive or backend” cognitive impairment assessment and therefore better collaborate with medical systems to promote early identification and treatment of mild cognitive impairment and dementia.

## Appendix

Multimedia Appendix 1 TRIPOD checklist: prediction model development and validation. TRIPOD: Transparent Reporting of a Multivariable Prediction Model for Individual Prognosis or Diagnosis.

Multimedia Appendix 2 Psychosocial and lifestyle questionnaire scales in the health and retirement study.

Multimedia Appendix 3 Description of missing values.

Multimedia Appendix 4 Training hyperparameter settings.

Multimedia Appendix 5 Sample characteristics for the training and testing datasets.

Multimedia Appendix 6 Comparison of AUC values between the seven candidate models in predicting current cognitive impairments. AUC: area under curve.

Multimedia Appendix 7 Predictive accuracy and performance of the assessment tool under different cost ratios of underdiagnosis to overdiagnosis.

Multimedia Appendix 8 Training and validation performance of the # MLP models in 10-fold cross-validation. MLP: multilayer perceptron.

Multimedia Appendix 9 Comparison of machine learning performance metrics after scale combination and inclusion of education level variable.

## Abbreviations

AUC: area under the curve
CHSW: community health and social worker
HRS: Health and Retirement Study
LQR: low-quality response
MCI: mild cognitive impairment
MLP: multilayer perceptron
TRIPOD: Transparent Reporting of a Multivariable Prediction Model for Individual Prognosis or Diagnosis

## Data Availability

The data and code that support the findings of this study are available from the corresponding authors on request.
