# Supplementary material for: Early Identification of Cognitive Impairment in Community Environments Through Modeling Subtle Inconsistencies in Questionnaire Responses: Machine Learning Model Development and Validation
Source: JMIR Form Res. 2024 Nov 13;8:e54335. doi: 10.2196/54335 (PMC11602764; doi:10.2196/54335)
Supplement: Multimedia Appendix 2 [file formative_v8i1e54335_app2.docx]

**Table S2**. Psychosocial and lifestyle questionnaire scales in the health and retirement study.

| **Index** | **Questionnaire name** | **Number of items** | **Response scale** |
| --- | --- | --- | --- |
| 1 | Life satisfaction | 5 | 6-point scale (strongly disagree – strongly agree) |
| 2 | Cynical hostility | 5 | 6-point scale (strongly disagree – strongly agree) |
| 3 | Optimism | 6 | 6-point scale (strongly disagree – strongly agree) |
| 4 | Hopelessness | 4 | 6-point scale (strongly disagree – strongly agree) |
| 5 | Loneliness | 3 | 3-point scale (often – hardly ever or never) |
| 6 | Neighborhood physical disorder | 4 | 7-point scale [positive vs negative descriptors as scale anchors, specific for each item] |
| 7 | Neighborhood social cohesion | 4 | 7-point scale [positive vs negative descriptors as scale anchors, specific for each item] |
| 8 | Constraints on personal control | 5 | 6-point scale (strongly disagree – strongly agree) |
| 9 | Perceived mastery | 5 | 6-point scale (strongly disagree – strongly agree) |
| 10 | Religiosity/Spirituality | 4 | 6-point scale (strongly disagree – strongly agree) |
| 11 | Everyday discrimination | 5 | 6-point scale (almost every day – never) |
| 12 | Social effort/reward balance | 3 | 5-point scale (strongly disagree – strongly agree) |
| 13 | Extraversion | 5 | 4-point scale (a lot – not at all) |
| 14 | Agreeableness | 5 | 4-point scale (a lot – not at all) |
| 15 | Neuroticism | 4 | 4-point scale (a lot – not at all) |
| 16 | Conscientiousness | 5 | 4-point scale (a lot – not at all) |
| 17 | Openness to experience | 7 | 4-point scale (a lot – not at all) |
| 18 | Purpose in life | 7 | 6-point scale (strongly disagree – strongly agree) |
| 19 | Anxiety | 5 | 4-point scale (never – most of the time) |
| 20 | Anger-in | 4 | 4-point scale (almost never – almost always) |
| 21 | Anger-out | 7 | 4-point scale (almost never – almost always) |
