# Supplementary material for: Early Identification of Cognitive Impairment in Community Environments Through Modeling Subtle Inconsistencies in Questionnaire Responses: Machine Learning Model Development and Validation
Source: JMIR Form Res. 2024 Nov 13;8:e54335. doi: 10.2196/54335 (PMC11602764; doi:10.2196/54335)
Supplement: Multimedia Appendix 3 [file formative_v8i1e54335_app3.docx]

**Table S3**. Description of missing values.

| No. | Item Name | Number of  participants with missing values | Proportion of  missing values |
| --- | --- | --- | --- |
| Life satisfaction | | | |
| 1 | In most ways my life is close to ideal. | 161 | 1.24% |
| 2 | The conditions of my life are excellent. | 188 | 1.45% |
| 3 | I am satisfied with my life. | 139 | 1.07% |
| 4 | So far, I have gotten the important things I want in life. | 133 | 1.03% |
| 5 | If I could live my life again, I would change almost nothing. | 132 | 1.02% |
| Optimism | | | |
| 1 | If something can go wrong for me it will. | 187 | 1.44% |
| 2 | I’m always optimistic about my future. | 170 | 1.31% |
| 3 | In uncertain times, I usually expect the best. | 132 | 1.02% |
| 4 | Overall, I expect more good things to happen to me than bad. | 98 | 0.76% |
| 5 | I hardly ever expect things to go my way. | 149 | 1.15% |
| 6 | I rarely count on good things happening to me. | 113 | 0.87% |
| Hopelessness | | | |
| 1 | I feel it is impossible for me to reach the goals that I would like to strive for. | 135 | 1.04% |
| 2 | The future seems hopeless to me and I can’t believe that things are changing for the better. | 128 | 0.99% |
| 3 | I don’t expect to get what I really want. | 105 | 0.81% |
| 4 | There’s no use in really trying to get something I want because I probably won’t get it. | 111 | 0.86% |
| Purpose in life | | | |
| 1 | I enjoy making plans for the future and working to make them a reality. | 154 | 1.19% |
| 2 | My daily activities often seem trivial and unimportant to me. | 165 | 1.27% |
| 3 | I am an active person in carrying out the plans I set for myself. | 151 | 1.17% |
| 4 | I don't have a good sense of what it is I'm trying to accomplish in life. | 210 | 1.62% |
| 5 | I sometimes feel as if I've done all there is to do in life. | 153 | 1.18% |
| 6 | I live life one day at a time and don’t really think about the future. | 142 | 1.10% |
| 7 | I have a sense of direction and purpose in my life. | 156 | 1.21% |
| Demographics | | | |
| 1 | age | 343 | 2.65% |
| 2 | gender | 343 | 2.65% |
