# Supplementary material for: Early Identification of Cognitive Impairment in Community Environments Through Modeling Subtle Inconsistencies in Questionnaire Responses: Machine Learning Model Development and Validation
Source: JMIR Form Res. 2024 Nov 13;8:e54335. doi: 10.2196/54335 (PMC11602764; doi:10.2196/54335)
Supplement: Multimedia Appendix 4 [file formative_v8i1e54335_app4.docx]

**Table S4**. Training hyperparameter settings.

| **Name** | **Configuration** |
| --- | --- |
| **Logistic Regression** | |
| C | 0.1/0.01 |
| Solver | lbfgs/saga |
| **Decision trees** | |
| Max_depth | 10 |
| Min_samples_split | 2/5 |
| **XGBoost** | |
| Learning_rate | 0.01/0.001 |
| N_estimators | 50/100 |
| **LightGBM** | |
| Learning_rate | 0.01/0.001 |
| N_estimators | 50/100 |
| **MLP** | |
| Hidden Layer Units | [200, 100, 50, 20] |
| Activation Function | ReLU |
| Output Activation | Sigmoid |
| Weight Initialization | he_normal |
| Dropout Rate | 0.2 ~ 0.3 |
| L2 Regularization | 0.01/0.001 |
| Optimizer | Adam (learning_rate=0.01) |
| Loss | Binary cross-entropy |
| Batch Size | 32/64 |
| ReduceLROnPlateau | factor=0.2, patience=2~5, min_learning_rate=0.0001 |
| Epochs | 300 |
| Early Stopping | patience=15~30, restore_best_weights=True |
| Class Weighting | cognitively normal = 0.61, cognitively impaired= 2.76 |
| **CNN-LSTM** | |
| Conv1D | (32,3) |
| MaxPooling1D | 2 |
| LSTM | 32 |
| **Bi-GRU** | |
| Bidirectional GRU (1st layer) | 32 |
| (2nd layer) | 16 |
