# Supplementary material for: Early Identification of Cognitive Impairment in Community Environments Through Modeling Subtle Inconsistencies in Questionnaire Responses: Machine Learning Model Development and Validation
Source: JMIR Form Res. 2024 Nov 13;8:e54335. doi: 10.2196/54335 (PMC11602764; doi:10.2196/54335)
Supplement: Multimedia Appendix 5 [file formative_v8i1e54335_app5.docx]

**Table S5**. Sample characteristics for the training and testing datasets.

| Characteristic | Training (n = 9059) | Testing (n = 3883) |  |
| --- | --- | --- | --- |
|  | **Frequency, *n* (%)** | **Frequency, *n* (%)** | ***P* value** |
| **Age in years** |  |  |  |
| 50 - 59 | 1828 (20.7) | 785 (20.9) | .98 |
| 60 - 69 | 2886 (32.7) | 1227 (32.6) | .79 |
| 70 - 79 | 2744 (31.1) | 1191 (31.7) | .68 |
| 80 and older | 1378 (15.6) | 560 (14.9) | .26 |
| **Gender** |  |  |  |
| Female | 5145 (58.2) | 2212 (58.8) | .87 |
| Male | 3691 (41.8) | 1551 (41.2) | .41 |
| **Marital Status** |  |  |  |
| Married | 5701 (64.5) | 2411 (64.1) | .38 |
| Not married | 3134 (35.5) | 1352 (35.9) | .82 |
| **Race** |  |  |  |
| White | 7440 (84.2) | 3148 (83.7) | .16 |
| African American | 1051 (11.9) | 460 (12.2) | .71 |
| Other | 345 (3.9) | 155 (4.1) | .66 |
| **Ethnicity** |  |  |  |
| Hispanic | 627 (7.1) | 276 (7.3) | .73 |
| Not Hispanic | 8209 (92.9) | 3487 (92.7) | .16 |
| **Education level** |  |  |  |
| High school and below | 4791 (54.2) | 2042 (54.3) | .77 |
| Some college | 2067 (23.4) | 851 (22.6) | .27 |
| College graduate and above | 1977 (22.4) | 870 (23.1) | .48 |
| **Self-reported diseases** |  |  |  |
| High blood pressure | 4908 (56.0) | 2121 (56.9) | .66 |
| Diabetes | 1669 (19.1) | 733 (19.7) | .56 |
| Heart disease | 2113 (24.2) | 850 (22.9) | .08 |
| Stroke | 681 (7.8) | 266 (7.2) | .19 |
| With two or more of the above diseases | 2640 (29.1) | 1088 (28) | .2 |
