# Supplementary material for: Early Identification of Cognitive Impairment in Community Environments Through Modeling Subtle Inconsistencies in Questionnaire Responses: Machine Learning Model Development and Validation
Source: JMIR Form Res. 2024 Nov 13;8:e54335. doi: 10.2196/54335 (PMC11602764; doi:10.2196/54335)
Supplement: Multimedia Appendix 6 [file formative_v8i1e54335_app6.docx]

**Table S6**. Comparison of AUC values between the seven candidate models in predicting current cognitive impairments. AUC: area under curve.

| **Index** | **Questionnaire name** | **MLP^b^** | **Logistic Regression** | **Decision Tree** | **XGBoost** | **LightGBM** | **Bi-GRU^c^** | **CNN-LSTM^d^** |
| --- | --- | --- | --- | --- | --- | --- | --- | --- |
| 1 | Life satisfaction | 0.63 (0.61 - 0.66)  /0.71 (0.69 - 0.73) | 0.61 (0.58 - 0.64)  /0.66 (0.64 - 0.68) | 0.56 (0.53 - 0.58  /0.61 (0.59 - 0.64) | 0.6 (0.57 - 0.62)  /0.71 (0.69 - 0.73) | 0.61 (0.58 - 0.63)  /0.7 (0.68 - 0.73) | 0.61 (0.59 - 0.63)  /0.71 (0.69 - 0.73) | 0.61 (0.59 - 0.63)  /0.7 (0.68 - 0.73) |
| 2 | Cynical hostility | 0.64 (0.61 - 0.66)  /0.73 (0.71 - 0.75) | 0.63 (0.61 - 0.65)  /0.69 (0.67 - 0.71) | 0.58 (0.55 - 0.6)  /0.63 (0.61 - 0.66) | 0.63 (0.6 - 0.65)  /0.71 (0.69 - 0.73) | 0.63 (0.61 - 0.66)  /0.72 (0.7 - 0.74) | 0.63 (0.61 - 0.65)  /0.72 (0.7 - 0.74) | 0.62 (0.6 - 0.64)  /0.71 (0.69 - 0.73) |
| 3 | Optimism | 0.66 (0.63 - 0.68)  /0.74 (0.72 - 0.76) | 0.65 (0.62 - 0.67)  /0.68 (0.65 - 0.7) | 0.58 (0.56 - 0.6)  /0.62 (0.6 - 0.65) | 0.64 (0.62 - 0.66)  /0.72 (0.7 - 0.74) | 0.64 (0.62 - 0.66)  /0.72 (0.7 - 0.74) | 0.64 (0.62 - 0.67)  /0.73 (0.71 - 0.75) | 0.64 (0.62 - 0.66)  /0.73 (0.71 - 0.75) |
| 4 | Hopelessness | 0.63 (0.61 - 0.65)  /0.71 (0.68 - 0.72) | 0.62 (0.59 - 0.64)  /0.66 (0.64 - 0.68) | 0.6 (0.57 - 0.62)  /0.67 (0.65 - 0.69) | 0.63 (0.6 - 0.65)  /0.71 (0.68 - 0.73) | 0.63 (0.6 - 0.65)  /0.71 (0.68 - 0.73) | 0.6 (0.58 - 0.63)  /0.72 (0.7 - 0.74) | 0.61 (0.59 - 0.64)  /0.7 (0.68 - 0.72) |
| 5 | Loneliness | 0.59 (0.56 - 0.61)  /0.69 (0.67 - 0.71) | 0.56 (0.54 - 0.59)  /0.67 (0.64 - 0.69) | 0.57 (0.55 - 0.6)  /0.64 (0.61 - 0.66) | 0.58 (0.55 - 0.6)  /0.68 (0.66 - 0.71) | 0.58 (0.56 - 0.61)  /0.69 (0.67 - 0.71) | 0.58 (0.56 - 0.61)  /0.69 (0.66 - 0.71) | 0.58 (0.56 - 0.61)  /0.7 (0.67 - 0.72) |
| 6 | Neighborhood physical disorder | 0.61 (0.59 - 0.63)  /0.7 (0.68 - 0.72) | 0.58 (0.56 - 0.6)  /0.69 (0.67 - 0.72) | 0.55 (0.53 - 0.57)  /0.62 (0.6 - 0.64) | 0.59 (0.57 - 0.62)  /0.68 (0.66 - 0.7) | 0.6 (0.58 - 0.62)  /0.7 (0.67 - 0.72) | 0.6 (0.58 - 0.62)  /0.69 (0.67 - 0.72) | 0.6 (0.58 - 0.63)  /0.69 (0.67 - 0.71) |
| 7 | Neighborhood social cohesion | 0.58 (0.55 - 0.6)  /0.7 (0.68 - 0.72) | 0.58 (0.55 - 0.6)  /0.7 (0.67 - 0.72) | 0.55 (0.53 - 0.58)  /0.63 (0.61 - 0.65) | 0.59 (0.56 - 0.61)  /0.69 (0.67 - 0.72) | 0.58 (0.55 - 0.6)  /0.69 (0.67 - 0.71) | 0.59 (0.56 - 0.61)  /0.7 (0.68 - 0.72) | 0.58 (0.55 - 0.6)  /0.7 (0.67 - 0.72) |
| 8 | Constraints on personal control | 0.64 (0.62 - 0.66)  /0.71 (0.69 - 0.73) | 0.64 (0.62 - 0.66)  /0.71 (0.69 - 0.73) | 0.58 (0.55 - 0.6)  /0.65 (0.63 - 0.67) | 0.64 (0.62 - 0.66)  /0.71 (0.69 - 0.73) | 0.63 (0.61 - 0.65)  /0.7 (0.68 - 0.72) | 0.62 (0.6 - 0.65)  /0.71 (0.69 - 0.74) | 0.64 (0.61 - 0.66)  /0.7 (0.68 - 0.72) |
| 9 | Perceived mastery | 0.57 (0.54 - 0.59)  /0.68 (0.66 - 0.7) | 0.56 (0.54 - 0.59)  /0.68 (0.66 - 0.7) | 0.53 (0.51 - 0.55)  /0.6 (0.57 - 0.62) | 0.57 (0.55 - 0.6)  /0.67 (0.65 - 0.69) | 0.57 (0.54 - 0.59)  /0.67 (0.65 - 0.69) | 0.58 (0.55 - 0.6)  /0.68 (0.66 - 0.7) | 0.55 (0.52 - 0.57)  /0.67 (0.65 - 0.69) |
| 10 | Religiosity/Spirituality | 0.56 (0.54 - 0.58)  /0.68 (0.65 - 0.7) | 0.56 (0.54 - 0.58)  /0.68 (0.66 - 0.7) | 0.57 (0.55 - 0.59)  /0.63 (0.61 - 0.66) | 0.57 (0.54 - 0.59)  /0.67 (0.65 - 0.69) | 0.56 (0.54 - 0.59)  /0.67 (0.65 - 0.69) | 0.56 (0.54 - 0.58)  /0.68 (0.65 - 0.7) | 0.56 (0.54 - 0.59)  /0.67 (0.65 - 0.7) |
| 11 | Everyday discrimination | 0.62 (0.6 - 0.64)  /0.71 (0.69 - 0.73) | 0.6 (0.57 - 0.62)  /0.64 (0.62 - 0.67) | 0.58 (0.56 - 0.61)  /0.64 (0.62 - 0.66) | 0.61 (0.58 - 0.63)  /0.71 (0.69 - 0.73) | 0.6 (0.58 - 0.62)  /0.7 (0.68 - 0.72) | 0.61 (0.58 - 0.63)  /0.7 (0.68 - 0.73) | 0.62 (0.6 - 0.64)  /0.71 (0.69 - 0.73) |
| 12 | Social effort/reward balance | 0.61 (0.58 - 0.63)  /0.69 (0.66 - 0.71) | 0.55 (0.52 - 0.57)  /0.63 (0.61 - 0.66) | 0.58 (0.55 - 0.6)  /0.62 (0.59 - 0.64) | 0.61 (0.59 - 0.63)  /0.68 (0.66 - 0.71) | 0.61 (0.59 - 0.64)  /0.69 (0.67 - 0.71) | 0.61 (0.59 - 0.63)  /0.68 (0.66 - 0.71) | 0.61 (0.59 - 0.63)  /0.69 (0.67 - 0.71) |
| 13 | Extraversion | 0.61 (0.59 - 0.63)  /0.71 (0.68 - 0.73) | 0.6 (0.58 - 0.63)  /0.7 (0.68 - 0.72) | 0.57 (0.54 - 0.59)  /0.61 (0.58 - 0.63) | 0.6 (0.58 - 0.63)  /0.7 (0.68 - 0.72) | 0.6 (0.57 - 0.62)  /0.7 (0.68 - 0.72) | 0.6 (0.58 - 0.62)  /0.71 (0.69 - 0.73) | 0.61 (0.58 - 0.63)  /0.7 (0.68 - 0.73) |
| 14 | Agreeableness | 0.6 (0.58 - 0.62)  /0.71 (0.69 - 0.73) | 0.6 (0.58 - 0.62)  /0.7 (0.68 - 0.73) | 0.53 (0.51 - 0.56)  /0.64 (0.61 - 0.66) | 0.58 (0.55 - 0.6)  /0.7 (0.68 - 0.72) | 0.6 (0.58 - 0.62)  /0.71 (0.68 - 0.73) | 0.6 (0.57 - 0.62)  /0.71 (0.69 - 0.73) | 0.59 (0.56 - 0.61)  /0.7 (0.68 - 0.72) |
| 15 | Neuroticism | 0.6 (0.57 - 0.62)  /0.7 (0.68 - 0.73) | 0.6 (0.58 - 0.62)  /0.71 (0.69 - 0.73) | 0.58 (0.55 - 0.6)  /0.6 (0.58 - 0.63) | 0.59 (0.57 - 0.62)  /0.7 (0.68 - 0.72) | 0.59 (0.57 - 0.62)  /0.7 (0.68 - 0.72) | 0.59 (0.57 - 0.61)  /0.7 (0.68 - 0.72) | 0.6 (0.58 - 0.63)  /0.7 (0.68 - 0.73) |
| 16 | Conscientiousness | 0.65 (0.63 - 0.68)  /0.72 (0.7 - 0.75) | 0.65 (0.63 - 0.68)  /0.72 (0.69 - 0.74) | 0.62 (0.59 - 0.64)  /0.64 (0.61 - 0.66) | 0.65 (0.63 - 0.67)  /0.71 (0.69 - 0.74) | 0.65 (0.62 - 0.67)  /0.71 (0.69 - 0.73) | 0.65 (0.63 - 0.67)  /0.72 (0.7 - 0.74) | 0.65 (0.63 - 0.68)  /0.72 (0.7 - 0.74) |
| 17 | Openness to experience | 0.63 (0.61 - 0.65)  /0.71 (0.69 - 0.74) | 0.62 (0.6 - 0.65)  /0.69 (0.66 - 0.71) | 0.56 (0.53 - 0.58)  /0.62 (0.6 - 0.65) | 0.62 (0.6 - 0.65)  /0.71 (0.69 - 0.74) | 0.63 (0.61 - 0.66)  /0.71 (0.69 - 0.73) | 0.63 (0.6 - 0.65)  /0.7 (0.68 - 0.72) | 0.62 (0.6 - 0.64)  /0.71 (0.69 - 0.73) |
| 18 | Purpose in life | 0.63 (0.61 - 0.65)  /0.71 (0.68 - 0.73) | 0.6 (0.58 - 0.63)  /0.7 (0.68 - 0.72) | 0.58 (0.56 - 0.61)  /0.62 (0.59 - 0.65) | 0.62 (0.59 - 0.64)  /0.69 (0.67 - 0.71) | 0.62 (0.6 - 0.64)  /0.69 (0.67 - 0.71) | 0.61 (0.58 - 0.63)  /0.7 (0.67 - 0.72) | 0.6 (0.58 - 0.63)  /0.7 (0.68 - 0.72) |
| 19 | Anxiety | 0.63 (0.61 - 0.65)  /0.71 (0.69 - 0.73) | 0.62 (0.6 - 0.65)  /0.71 (0.69 - 0.73) | 0.58 (0.56 - 0.61)  /0.64 (0.62 - 0.67) | 0.62 (0.6 - 0.64)  /0.7 (0.68 - 0.72) | 0.61 (0.59 - 0.64)  /0.7 (0.67 - 0.72) | 0.62 (0.6 - 0.64)  /0.7 (0.68 - 0.73) | 0.63 (0.61 - 0.65)  /0.71 (0.69 - 0.73) |
| 20 | Anger-in | 0.58 (0.55 - 0.6)  /0.68 (0.66 - 0.7) | 0.56 (0.53 - 0.58)  /0.62 (0.6 - 0.64) | 0.57 (0.55 - 0.6)  /0.63 (0.6 - 0.65) | 0.59 (0.56 - 0.61)  /0.68 (0.65 - 0.7) | 0.59 (0.57 - 0.61)  /0.68 (0.66 - 0.7) | 0.59 (0.56 - 0.61)  /0.67 (0.65 - 0.69) | 0.58 (0.56 - 0.6)  /0.68 (0.66 - 0.71) |
| 21 | Anger-out | 0.57 (0.54 - 0.59)  /0.69 (0.66 - 0.71) | 0.55 (0.53 - 0.58)  /0.68 (0.66 - 0.7) | 0.55 (0.53 - 0.58)  /0.63 (0.61 - 0.66) | 0.59 (0.56 - 0.61)  /0.68 (0.65 - 0.7) | 0.58 (0.56 - 0.6)  /0.68 (0.66 - 0.7) | 0.58 (0.56 - 0.61)/  0.69 (0.67 - 0.71) | 0.55 (0.53 - 0.58)/  0.69 (0.66 - 0.71) |

^a^AUC: area under the curve (with 95% confidence interval). Each pair of values represents the AUC values for each model, where the first value represents the predictor as the LQR index only, and the second value represents the predictor as the LQR indices plus age and gender.

^b^MLP: multilayer perceptron

^c^Bi-GRU: bidirectional-gated recurrent unit

^d^CNN-LSTM: convolutional neural network-long short-term memory
