# Supplementary material for: Early Identification of Cognitive Impairment in Community Environments Through Modeling Subtle Inconsistencies in Questionnaire Responses: Machine Learning Model Development and Validation
Source: JMIR Form Res. 2024 Nov 13;8:e54335. doi: 10.2196/54335 (PMC11602764; doi:10.2196/54335)
Supplement: Multimedia Appendix 7 [file formative_v8i1e54335_app7.docx]

**Multimedia Appendix 7**

Predictive accuracy and performance of the assessment tool under different cost ratios of underdiagnosis to overdiagnosis.

| Model | AUC^a^ | Threshold cost ratio=1 | Referred for further assessment, n (%) | Cognitive impairment identified, n (%) | Threshold cost ratio=2 | Referred for further assessment, n (%) | Cognitive impairment identified, n (%) | Threshold cost ratio=4 | Referred for further assessment, n (%) | Cognitive impairment identified, n (%) | Threshold cost ratio=6 | Referred for further assessment, n (%) | Cognitive impairment identified, n (%) |
| --- | --- | --- | --- | --- | --- | --- | --- | --- | --- | --- | --- | --- | --- |
| LQR^b^ indices from optimism scale | 0.66 (0.63-0.68) | 0.85 | 0 (0) | 0 (0) | 0.73 | 116 (3) | 49 (7) | 0.5 | 1553 (39.99) | 422 (59.9) | 0.42 | 2058 (53) | 507 (72) |
| LQR indices from purpose in life scale | 0.63 (0.61-0.65) | 0.92 | 0 (0) | 0 (0) | 0.71 | 78 (2) | 35 (5) | 0.52 | 1709 (44.01) | 422 (59.9) | 0.43 | 2407 (61.99) | 535 (76) |
| LQR indices from hopelessness scale | 0.63 (0.61-0.65) | 0.92 | 0 (0) | 0 (0) | 0.92 | 0 (0) | 0 (0) | 0.53 | 1281 (32.99) | 338 (48) | 0.43 | 2407 (61.99) | 542 (77) |
| LQR indices from life satisfaction scale | 0.63 (0.61-0.66) | 0.89 | 0 (0) | 0 (0) | 0.64 | 155 (4) | 49 (7) | 0.47 | 1631 (42) | 408 (58) | 0.46 | 1747 (44.99) | 436 (61.9) |
| LQR indices from optimism scale plus age and gender | 0.74 (0.72-0.76) | 0.74 | 39 (1) | 35 (5) | 0.65 | 388 (10) | 169 (24) | 0.53 | 1359 (35) | 444 (63.1) | 0.45 | 1903 (49.01) | 542 (77) |
| LQR indices from purpose in life scale plus age and gender | 0.71 (0.68-0.73) | 0.92 | 0 (0) | 0 (0) | 0.64 | 699 (18) | 260 (37) | 0.55 | 1281 (32.99) | 422 (59.9) | 0.43 | 2097 (54) | 549 (78) |
| LQR indices from hopelessness scale plus age and gender | 0.71 (0.68-0.72) | 0.79 | 78 (2) | 35 (5) | 0.67 | 388 (10) | 169 (24) | 0.5 | 1553 (39.99) | 458 (65.1) | 0.44 | 2136 (55.01) | 556 (79) |
| LQR indices from life satisfaction scale plus age and gender | 0.71 (0.69-0.73) | 0.73 | 116 (3) | 56 (8) | 0.6 | 777 (20) | 289 (41.1) | 0.54 | 1243 (32.01) | 408 (58) | 0.46 | 1786 (46) | 507 (72) |

^a^AUC: area under the curve (with 95% CI).

^b^LQR: low-quality response.
