# Supplementary material for: Early Identification of Cognitive Impairment in Community Environments Through Modeling Subtle Inconsistencies in Questionnaire Responses: Machine Learning Model Development and Validation
Source: JMIR Form Res. 2024 Nov 13;8:e54335. doi: 10.2196/54335 (PMC11602764; doi:10.2196/54335)

**Figure S8**. Training and validation performance of the # MLP models in 10-fold cross-validation. MLP: multilayer perceptron.


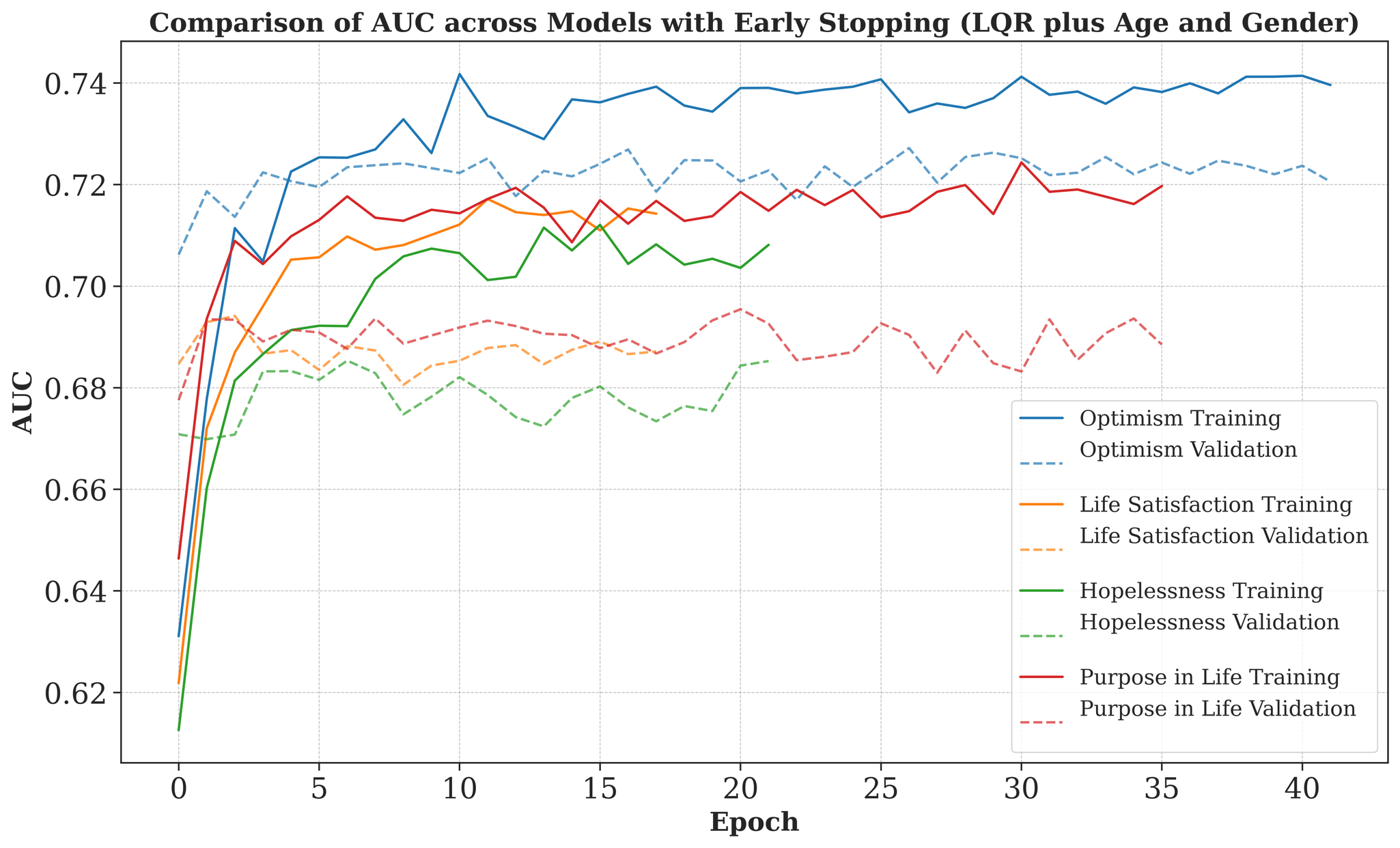


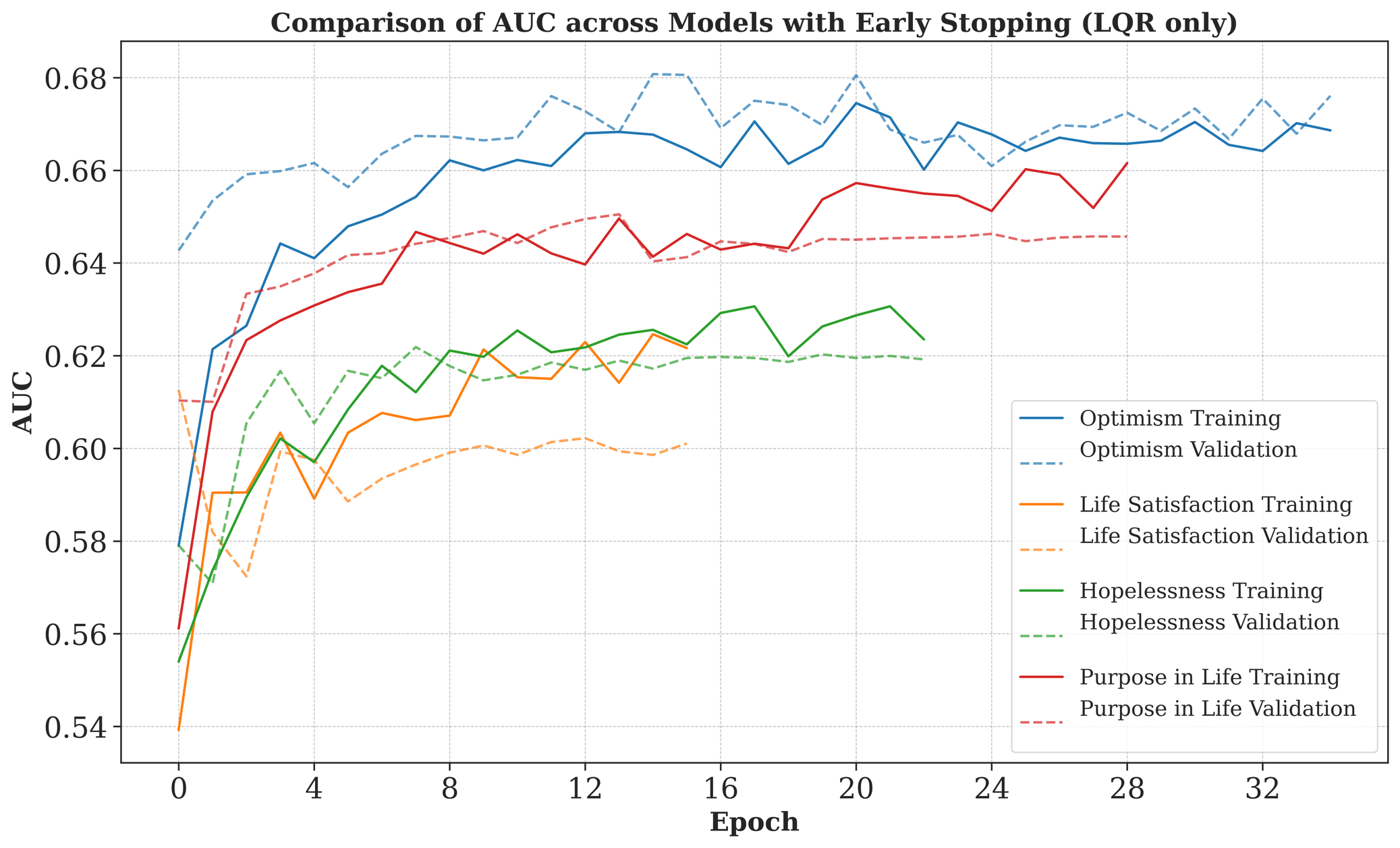

Supplement: Multimedia Appendix 8 [file formative_v8i1e54335_app8.docx]
