# Supplementary material for: Early Identification of Cognitive Impairment in Community Environments Through Modeling Subtle Inconsistencies in Questionnaire Responses: Machine Learning Model Development and Validation
Source: JMIR Form Res. 2024 Nov 13;8:e54335. doi: 10.2196/54335 (PMC11602764; doi:10.2196/54335)
Supplement: Multimedia Appendix 9 [file formative_v8i1e54335_app9.docx]

**Table S9**. Comparison of machine-learning performance metrics after scale combination and inclusion of education level variable.

| **Model** | **AUC** | **Sensitivity** | **Specificity** |
| --- | --- | --- | --- |
| Optimism | | | |
| LQR only | 0.66 (0.63-0.68) | 0.6 | 0.63 |
| LQR_age_gender | 0.74(0.72-0.76) | 0.64 | 0.7 |
| LQR_age_gender_educ_level | 0.80 (0.78-0.81) | 0.75 | 0.7 |
| Purpose in Life | | | |
| LQR only | 0.63 (0.61-0.65) | 0.62 | 0.59 |
| LQR_age_gender | 0.71(0.68-0.73) | 0.6 | 0.72 |
| LQR_age_gender_educ_level | 0.79 (0.77-0.81) | 0.72 | 0.73 |
| Hopelessness | | | |
| LQR only | 0.63 (0.61-0.65) | 0.51 | 0.69 |
| LQR_age_gender | 0.71(0.68-0.72) | 0.65 | 0.66 |
| LQR_age_gender_educ_level | 0.79 (0.77-0.81) | 0.68 | 0.77 |
| Life Satisfaction | | | |
| LQR only | 0.63 (0.61-0.66) | 0.58 | 0.62 |
| LQR_age_gender | 0.71(0.69-0.73) | 0.58 | 0.73 |
| LQR_age_gender_educ_level | 0.79 (0.77-0.81) | 0.69 | 0.74 |
| Combination of four questionnaires | | | |
| LQR only | 0.69 (0.66-0.71) | 0.71 | 0.57 |
| LQR_age_gender | 0.75 (0.73-0.77) | 0.77 | 0.61 |
| LQR_age_gender_educ_level | 0.79 (0.77-0.80) | 0.74 | 0.71 |
